# On-Site X-ray Fluorescence Spectrometry Measurement Strategy for Assessing the Sulfonation to Improve Chemimechanical Pulping Processes

**DOI:** 10.1021/acsomega.2c07086

**Published:** 2022-12-15

**Authors:** Hafizur Rahman, Siwen An, Börje Norlin, Erik Persson, Per Engstrand, Faisal Zeeshan, Thomas Granfeldt, Tomáš Slavíček, Gunilla Pettersson

**Affiliations:** † Mid Sweden University, Holmgatan 10, 851 70 Sundsvall, Sweden; ‡ MAX IV Laboratory, Lund University, 225 91 Lund, Sweden; § Valmet AB, 851 94 Sundsvall, Sweden; ∥ Institute of Experimental and Applied Physics, Czech Technical University, Husova 240/5, 11000 Prague, Czech Republic

## Abstract

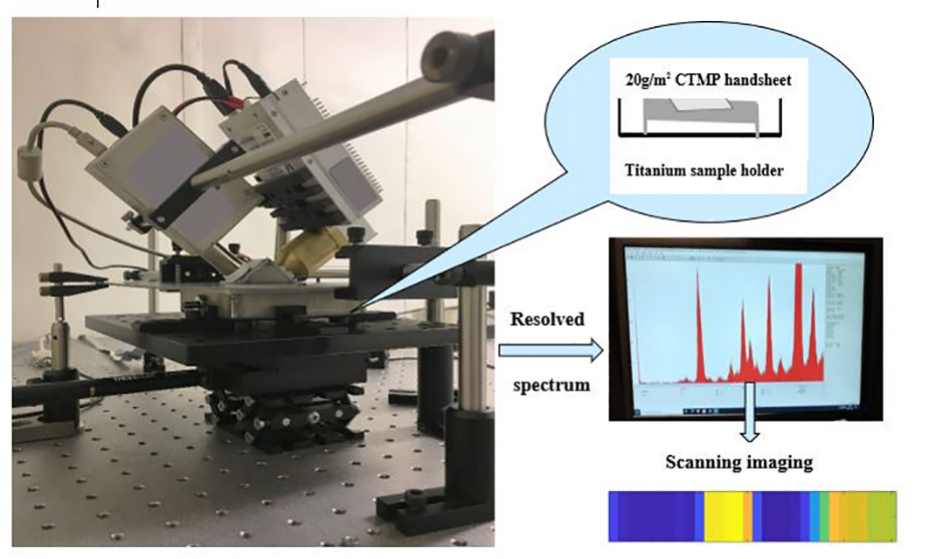

Minimizing the fiber property distribution would have the potential to improve the pulp properties and the process efficiency of chemimechanical pulp. To achieve this, it is essential to improve the level of knowledge of how evenly distributed the sulfonate concentration is between the individual chemimechanical pulp fibers. Due to the variation in quality between pulpwood and sawmill chips, as well as the on-chip screening method, it is difficult to develop an impregnation system that ensures the even distribution of sodium sulfite (Na_2_SO_3_) impregnation liquid. It is, therefore, crucial to measure the distribution of sulfonate groups within wood chips and fibers on a microscale. Typically, the degree of unevenness, i.e., the amount of fiber sulfonation and softening prior to defibration, is unknown on a microlevel due to excessively robust or complex processing methods. The degree of sulfonation at the fiber level can be determined by measuring the distribution of elemental sulfur and counterions of sulfonate groups, such as sodium or calcium. A miniaturized energy-dispersive X-ray fluorescence (ED-XRF) method has been developed to address this issue, enabling the analysis of sulfur distributions. It is effective enough to be applied to industrial laboratories for further development, i.e., improved image resolution and measurement time.

## Introduction

The production of chemimechanical pulp (CMP/CTMP/HTCTMP) has increased significantly in recent years for the production of hygiene products and even more so for packaging materials, such as paperboard and liner. In addition to the growing trend toward replacing plastics with renewable packaging materials that are easily recyclable and compostable, such as those produced by the paper industry, there is also an increased demand to improve the fundamental scientific understanding of pulp and paper manufacturing systems. As pulp is produced from chips, regardless of whether it is a high-yield pulping process (chemimechanical or semichemical) or chemical pulping process (kraft or sulfite), the efficiency of impregnation is crucial.^1^ It has always been very challenging to study the degree of effectiveness from the earlier suggested impregnation improvements.^2−8^

The chemithermomechanical pulp (CTMP) process, pretreatment of wood chips before defibration constitutes the core aspect of CTMP production. By increasing force in the direction of wood fibers during wood chipping, the wood structure is opened up, increasing the efficiency of CTMP impregnation.^9,10^ The minimum amount of electric energy needed for the separation of wood into individual fibers is directly related to the softening of lignin. In CTMP, wood chips soften as a function of temperature in a preheater and in a refiner, where fiber separation is achieved. The wood chips at CTMP are steamed, followed by compression in a plug screw; the chips are allowed to expand in an impregnation liquor such as sodium sulfite (Na_2_SO_3_) that lower the temperature than the steamed wood chips.^1,11−14^ Based on the study, the softening temperature of black eastern spruce decreased by about 2 °C when the sulfur content increased by 0.1% (as Na_2_SO_3_ equivalents), while sulfur (as Na_2_SO_3_ equivalents) content ranged from 0.3 to 2.8%.^15^ In the CTMP process, the softening process is accomplished using sodium sulfite (Na_2_SO_3_) solution at high temperatures (130–180 °C) during preheating and refining. A key mechanism behind the sulfonation of wood is to reduce the number of cross-links in the lignin by breaking covalent bonds when sulfite ions react with α carbon in a coniferyl alcohol structure.^16^ The structural change makes the wood softer at a certain temperature.^15,17,18^ Earlier studies have examined the effects of sulfite concentration and temperature on wood sulfonation kinetics at pH 9.^19^ Under CTMP conditions, sulfonation shifts fiber separation toward the middle lamella so that primary cell walls and middle lamellas are mainly separated.^15,20^ It is because lignin is unevenly distributed within the fiber cell wall, and concentrations are higher in the middle lamella that a change in dynamic modulus will affect fracture mechanics during refining.^21^ However, the combination of refining intensity and chemical softening by means of sulfonation and temperature-assisted softening during chip refining has already been examined with full-scale trials and implementations at the Holmen Braviken paper mill, Sweden.^22^ The basic strategy of sulfonation for CTMP is to obtain the minimum possible shive content and well-separated fibers and thus linked to increasing the wood softening before defibration in a chip refiner. It has been studied that the feasibility of softening chips to produce CTMP that has well-preserved fibers with yields above 95%, shives content less than 1% before the screening, and energy consumption less than 200 kWh/h.^23^

In the CTMP process, there is a key challenge in achieving even chemical distribution of sodium sulfite (Na_2_SO_3_) and sodium hydroxide (NaOH) inside the wood chips, especially for the hardwoods due to the different sizes of the wood chips: length (∼20 mm), thickness (∼3 to 4 mm), and fiber width (20–40 μm) and length (1.5–5 mm) of Norway spruce. Previous scanning transmission electron microscopy-energy-dispersive spectrometry (STEM-EDS) studies on sulfonated wood chips of southern pines found variability in sulfur distributions in cell walls after the treatment of alkaline sodium sulfite.^24^ During the impregnation process, the inner parts of the wood chips accept a much lower degree of sulfonation than the outer parts. A wood chip with less sulfonation or an unsulfonated chip tends to fracture in the outer secondary cell wall leaving a carbohydrate-rich fiber surface with different bonding characteristics.^25,26^ Increasing the sulfonation of the secondary wall increases the sheet density and strength, as well as the flexibility and conformability of fiber walls.^24^ This uneven sulfonation is one of the factors that effects on pulp properties with higher shive content. By manipulating the degree of sulfonation of each wall layer, pulp properties could be controlled to meet specific end uses. However, to develop selective chemical impregnation methods for chemithermomechanical pulping (CTMP), fast and accurate measurements are therefore necessary. Due to the lack of fast measurements, it is currently difficult to optimize impregnation processes. Currently, there is no specific method for measuring the sulfonate distribution in wood chips and individual fibers at the microscale level. If there were, it would enable better knowledge of sulfonation before defibration, thereby perhaps making the manufacturing of CTMP and neutral sulfite semichemical (NSSC) more even and effective. However, the precise degree of unevenness, i.e., the degree of sulfonation and softening of each fiber in the chip refiner before being defibrated, remains largely unknown due to the complicated procedures used.

Although this sulfonation of CTMP research is of interest, few studies have been conducted earlier. Since the distribution of sulfur requires extensive measurements, it is costly to conduct in detail. In earlier studies, microtone cut layers of 100 μm wood chips were used to determine the sulfonation degree (sulfur content of the washed samples) and impregnation efficiency (total wavelength of the unwanted samples) of individual wood chips.^19,21,27^ Sulfur concentrations were then determined via Schöniger combustion after dissolving wood chips in acid. Therefore, the presence of sulfur as sulfate (SO_4_^2–^) ions was found whose content was determined using ion chromatography.^19,21^ There was another CTMP study on birch wood blocks at various conditions.^12^ In that study, the distribution of sulfur longitudinally was studied at various points using a scanning electron microscope (SEM) equipped with energy-dispersive X-rays. With such a method, sulfur and other counterion such as sodium concentrations appeared to be related to measurements of sulfur by ion chromatography and sodium by atomic absorption spectrometry. At both ends of the woodblock, the amount of sulfite (SO_3_^2–^) was almost the same as expected, but it was reduced in the middle layer of wood.^12^ Meanwhile, various analytical techniques can be employed to derive an elemental distribution map in different research disciplines. Some examples include X-ray absorption spectroscopy, X-ray fluorescence (XRF) spectroscopy, and scanning electron microscopy and energy-dispersive X-ray spectroscopy (SEM-EDS).^28−34^ The research center Sensible Things that Communicate (STC) of Mid Sweden University, Sweden, has already been investigated at a laboratory-scale setup utilizing XRF on the coating of paperboard to simultaneously measure calcium (Ca) (3.7 keV) mapping and target copper (Cu) (8.0 keV) behind the paper.^33^ By utilizing high-resolution measurements of sulfur distribution, we also anticipate that modern miniature X-ray-based techniques will contribute to the study of the sulfonation degree on-site impregnation process of CTMP.

This study suggests the use of a miniaturized, energy-dispersive X-ray fluorescence (ED-XRF) scan using a collimated X-ray source and utilizing a spectroscopy method based on energy-dispersive X-rays. Detection of light elements at miniaturized ED-XRF setups was challenging at first due to low fluorescence yields and air absorption from uneven wood chip surfaces. It was suggested that impregnated pulp samples or paper samples with a plain surface can be used instead. For this reason, we placed the paper sample produced from unbleached CTMP in a titanium (Ti) box in an atmosphere of air and helium (He). In a one-line measurement, the sample surface was scanned in a few steps, resulting in the production of an elemental distribution image of the substances as photon counts. Our study showed that it is possible to improve the setup by increasing the fluorescence yield for the study of the degree of sulfonation in chip refining of CTMP at an industrial lab scale. Our hypothesis is that the efficiency and evenness of fiber separation in the chip refiner are highly dependent on how evenly the chips have been sulfonated. We believe that a more even sulfonation distribution can significantly reduce the specific energy demand in chip refining for certain shive contents.

However, we are investigating and establishing the following precise on-site XRF methodology for validating whether the direct X-ray fluorescence method can be used to measure the distribution of the sulfonate group in CTMP paper samples ranging in size from a few millimeters (mm) to a micrometer (μm).

## Methods and Materials

### Principle of XRF

X-ray fluorescence (XRF) is a technique for determining the elemental composition of a material by measuring the emission of characteristic fluorescent X-rays generated by a high-energy X-ray beam. By analyzing the photons emitted, elements can be determined to be present.^35^

### Instrumental Setup

**Figure 1:**
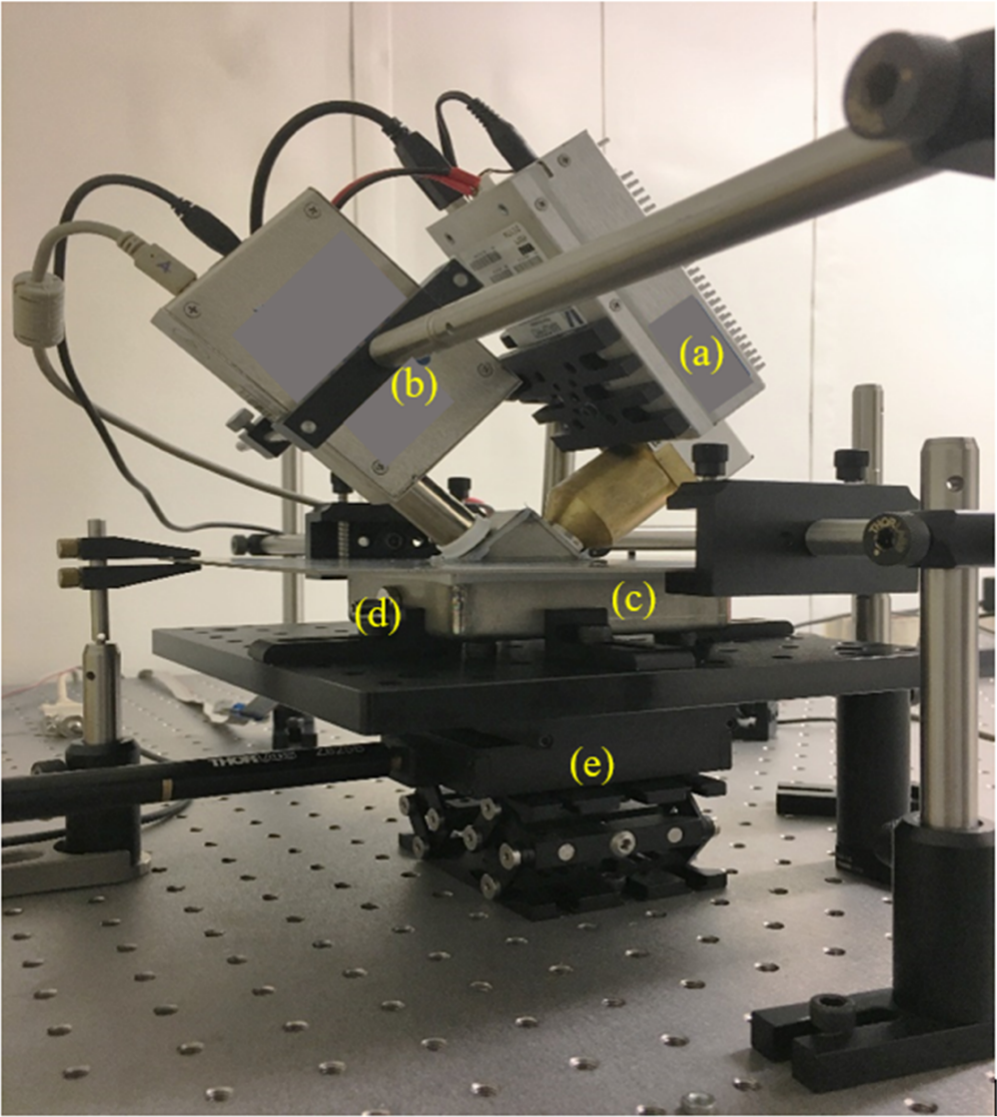
XRF measurement setup before being placed into a stainless-steel box.

In the XRF experiment, the sample was scanned to obtain the spectrum of a single-spot size, and then, the element of an interest peak was extracted and reconstructed by producing a spectrum of the element of interest. Using a collimated X-ray source and spectroscopic detector, this micro-X-ray technology can produce an elemental spectrum of sulfur and possibly other counterion such as sodium across wood chips or individual fibers in paper samples. An image of our XRF-based miniature setup is shown in Figure 1. To maintain the helium gas environment, a titanium cover palate was affixed to the titanium shield box. There was a 1.6 mm thick titanium plate with a 99.2% purity (metal basis). A two-dimensional (2D) stepper motor unit (Thorlabs) was connected to the sample box. To scan the sample in two dimensions, the stepper motor traveled a distance of 25 mm, and the minimum step size was 0.05 μm. An X-ray tube (Moxtek, 60 kV 12 W MAGPRO) and a spectrometer (Amptek X-123SDD) were mounted in air. The entire setup was covered with lead (Pb) sheets (thickness 1 mm, supplied by Nuclear Shields) into stainless-steel boxes to prevent radiation leakage.

The figure shows (a) a MOXTEK X-ray source, (b) an AMETEK spectrometer used to measure the spectrum of elements, (c) a 10 × 10 × 2.5 cm^3^ titanium shield box to hold the sample at an helium gas atmosphere, (d) helium gas connection, and (e) a stepper motor 2D (*XY* directional movement).

The focal spot of an X-ray tube is typically 400 μm, and the beam diverges from its source. As a result, a large area of the sample was being used, and the fluorescence photons were accumulating in the spectrometer. To improve the XRF scanning image resolution, the X-ray was collimated using a pinhole to reduce the focus spot size. To obtain a scanning image of individual fibers, the beamline focal point needs to be as small as a few micrometers. An XRF setup was carried out with three-pinhole collimators. The two handmade pinholes had diameters of 10 and 50 μm made of tungsten carbide (WC, Alfa Aesar, 99.95% pure metal) that had shape defects in comparison to a commercial pinhole with a diameter of 100 μm made of gold (Au) and platinum (Pt). A pinhole reduces the beam intensity and increases the measurement time since only a portion of the X-ray passes through the collimator. Spot-scan imaging was employed using two-dimensional precision translation stages, each with a 25 mm travel distance (Thorlabs).

### Sample Preparation

Valmet’s CTMP-712 pilot unbleached washed pulp was chosen for sulfur distribution to validate the XRF method. As the CTMP was thoroughly washed, there should be no other sulfur except for covalently bonded sulfur. The strategy was to estimate the differences in sulfur photon counts at high and low proportions of CTMP pulp from different handsheets. CTMP was diluted with bleached softwood kraft pulp (BSWK) of SCA reference kraft K44. An unbeaten low grammage handsheet of 20 gm/m^2^ was measured for single line scanning in this setup. The low grammage handsheet 20 gm/m^2^ was prepared by mixing CTMP and BSWK at different percentages according to the ISO 5269-2:2004 standard with tap water using a conventional sheet former with a surface area of 0.021 m^2^ at the SCA R&D Centre in Sundsvall, Sweden.^36^ For the study of sulfonation degree, four different proportions as 100% CTMP, 50% CTMP + 50% BSWK, 30% CTMP + 70% BSWK, and 20% CTMP + 80% BSWK were used.

Earlier studies reported a significantly lower sulfur and sodium circulation system in unbleached kraft pulp, and after bleaching, the total sulfur output and input were the same as those detected by the WinMOPS system in the Metso paper mill.^37^ Since unbleached kraft (UBK) pulps with some lignin are thoroughly washed, bleached softwood kraft (BSWK) should not contain any residual sulfur unlike unbleached kraft pulps. It is also expected that the counterion sodium concentration would be too low to detect as well for kraft pulp. Therefore, bleached kraft pulp was mixed with unbleached CTMP pulp at different percentages to dilute the CTMP’s sulfur content. However, fewer sulfur photons were expected gradually by decreasing the percentage of CTMP pulp consistency in these handsheets for the experiment.

Considering the measurement time, a higher concentration of sulfur and sodium was necessary to detect fluorescence photons from light elements. In contrast to sulfur, S (Kα_1_ 2.31 keV), sodium, Na (Kα_1_ 1.04 keV), was challenging to detect with the CTMP handsheet because of its low fluorescence yield. Seltin salt was used as an additional sample for the setup validation since it contains a high concentration of sodium and sulfur compared with other elements. Seltin salt, which is manufactured by Cederroth International AB, contains 50 g of NaCl (per 100 g) as well as KCl, MgSO_4_, and I.^38^

## Results and Discussion

### X-ray Attenuation

**Figure 2:**
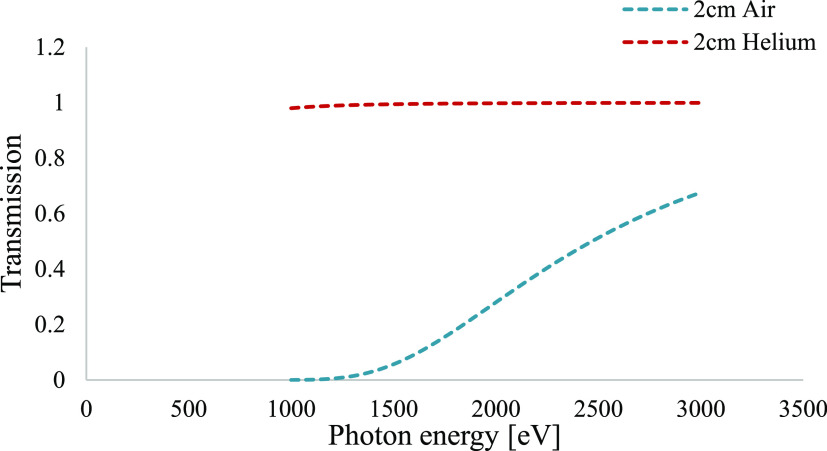
Calculated photon transmission in 2 cm air and helium.

Upon excitation of the XRF, fluorescence photons must pass through the media and reach the spectrometer. Fluorescence X-ray radiation from the light element has relatively low energy (long wavelength), and it is severely attenuated by air. Before the XRF setup, it was simulated using a Monte Carlo-*N*-particle radiation transport code (MCNP).^39^ Using X-ray-oriented programs (XOPs), photon transmission curves in air and helium gas relevant for light elements were plotted and can be seen in Figure 2. The simulation assumes 2 cm thickness of air or helium. For air, it results in a lowering of the sulfur peak at 2.31 keV and probably no sodium peak at all was left at 1.04 keV. Helium gas is therefore necessary for the detection of sodium, while sulfur can also be possible to detect in air although the signal decreases. It is possible to think of a vacuum atmosphere as an ideal situation in which there is no air absorption, but the difficulties associated with the maintenance of moving parts in a vacuum have major implications for the design of the instrument at a laboratory scale. For this application, a helium gas chamber is sufficient for measuring sodium.

In this study, a titanium box in a helium gas environment was designed to minimize the fluorescence photon absorption rather than a vacuum environment. Air environment was also considered here, but shield scatter photons in the air interfered with the measurements.

### Validation of Feasibility of XRF Setup

**Figure 3:**
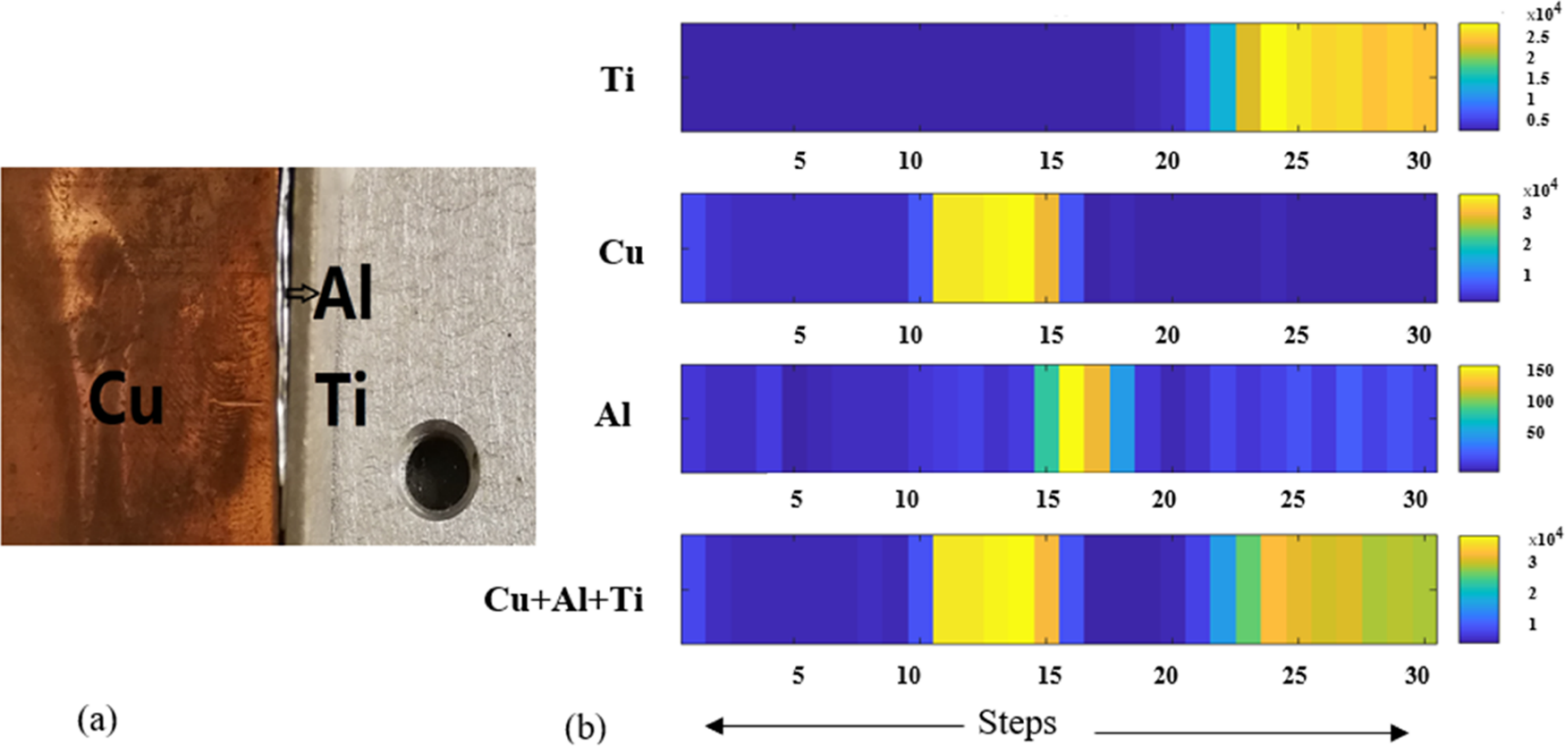
(a) Image of the Al foil set between Ti and Cu plate. (b) As shown in the figure, Al, Cu, and Ti elements are distributed across X, which are the scanning steps. For each element, the color bar shows the signal intensity.

To investigate the imaging system resolution of an XRF setup, it is necessary to choose the right pinhole for light elements with small sample sizes. In the air, a preliminary measurement of 60 μm of aluminum (Al) foil was performed. A sandwich structure is shown in Figure 3a, where the aluminum foil is sandwiched between copper and titanium plates. For primary verification of feasibility in the XRF setup, the 80 μm step size of one-line scanning measurement was used to determine the element distribution of Al, Cu, and Ti. A total of 30 steps were scanned. Using a 100 μm pinhole at an X-ray source, the measurement tube setting was 15 keV for 20 min for each step. The photon counts of the characteristic peaks of Al, Cu, and Ti in all measured spectra were integrated. The element distribution map for Al, Cu, and Ti can be seen in Figure 3b.

A fluorescence peak for aluminum appears at 1.48 keV (Kα_1_) and 1.56 keV (Kβ_1_). As the spectrometer has a 125 eV full width at half-maximum (FWHM), it cannot distinguish these aluminum peaks. Therefore, they merge into one peak in the output spectrum. Copper has a characteristic peak at 8.04 keV (Kα_1_), and Ti has a peak at 4.51 keV (Kα_1_), which is heavier than Al. A higher photon count corresponds to a higher concentration in the color bar. To draw elemental maps, we, therefore, combined spectral information with representative positions. As a result, the maps are in agreement with the sample structure. Due to the air absorption of the Al signal and the low fluorescence yield, the order of magnitude of photon counts from aluminum is much lower than those from titanium and copper.

**Figure 4:**
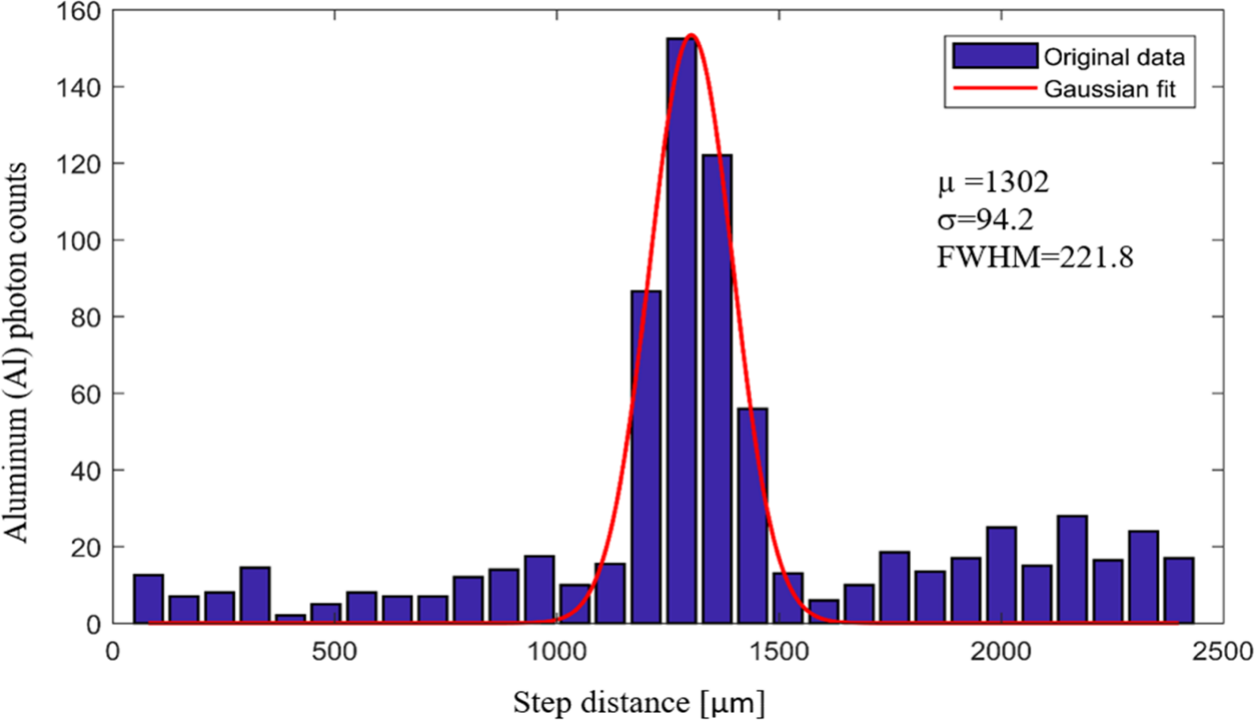
Investigation of the imaging system of the XRF setup using Gaussian fitting of the Al signal.

To test the imaging system resolution of the XRF setup, the histogram of the aluminum signal with Gaussian fitting is shown in Figure 4. According to XRF images, for a 60 μm aluminum foil, the full width at half-maximum (FWHM) was 221.8 μm due to the pinhole size and magnification. Using an 80 μm scan step size, overlapped scanning was used. In scanning measurements, the spatial resolution of the XRF image is limited by the spot size of the source and the scanning step size. In this setup, a pinhole is used to collimate the size of the source beam. As a result of the geometry magnification and pinhole diameter, the size of the focused spot became relatively large for our application. The size of the focused spot can be reduced by reducing the pinhole to a sample distance while keeping the pinhole diameter, by introducing polycapillary optics or by scanning with overlap. A previous study reported the spatial resolution of the XRF image scanning pitch and the mode of scanning (stepwise or continuous) where the 50% overlap (the pitch size is half the focal spot size) is usually considered a possibility to improve spatial resolution.^40,41^

### Validation of Helium Gas in an XRF Setup

**Figure 5:**
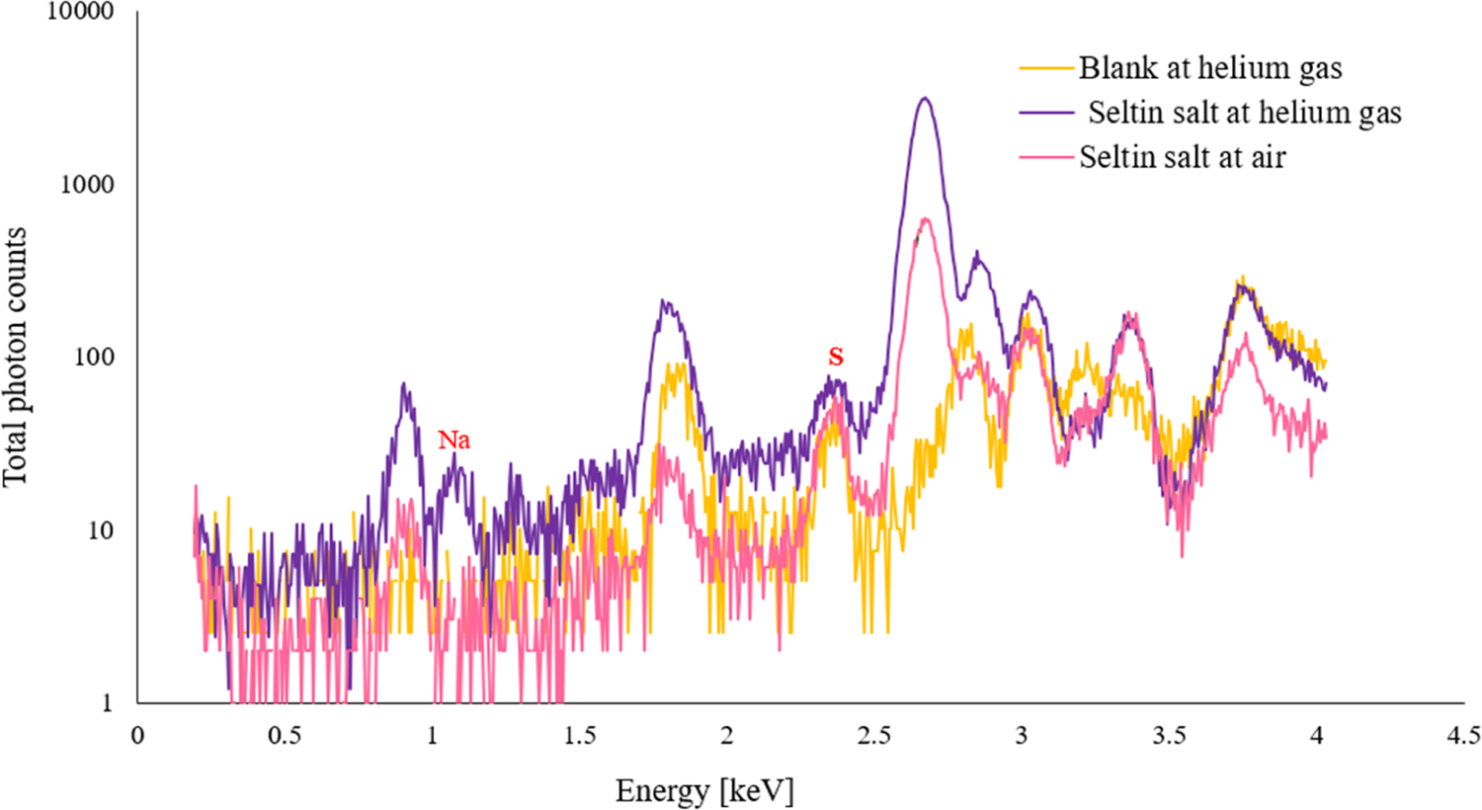
Sodium and sulfur peaks in Seltin salt under an helium gas environment using a 10 μm pinhole.

Following the investigation of X-ray attenuation and verification of feasibility of the XRF setup, it was further investigated on Seltin salt using a 10 μm pinhole at 8 keV fluorescence energy in a helium gas environment. In air and helium gas environment, a comparable peak of sodium (Kα_1_ 1.04 keV) and sulfur (Kα_1_ 2.31 keV) was observed; see Figure 5. In Seltin salt, the mass fraction for sodium was calculated to be 196.6 g/kg. This is considered a high sodium concentration. However, it resulted in a detection rate of sodium fluorescence photons of only 28 counts in 17 h and thus indicated that the 10 μm pinhole greatly reduced beam intensity. As defined by the American Chemical Society (ACS), the limit of detection (LOD) is a concentration above SNR ≥ 3, which means the characteristic peak of an element at a given concentration exceeds the background by a statistically significant amount. We assume a linear relationship between the photon counts and the concentration in the XRF spectrum. Thus, the LOD for this setup was 29 g/kg in which a 10 μm pinhole was used.

According to earlier studies using a semiparametric neutron activation analysis, the sodium concentration in the bright paper was about 2 g/kg, which was lower than the LOD of this setup.^42^ It is difficult to measure sodium in an XRF analysis because of its low concentration, low fluorescence yield, air absorption, and small pinhole. Moreover, we found that the Mα_1_-line from lead (Pb), interfering with the sulfur Kα_1_ at 2.34 keV exists in the blank’s measurement from lead (radiation shielding material outside the titanium box), and it interfered with the detection of sulfur in the Seltin salt. For the next real sample measurement of the CTMP and BSWK (50:50) handsheet, we shielded the X-ray tube with an aluminum pipe to avoid lead interference.

### XRF Setup in Both Helium Gas and Air Environments

**Figure 6:**
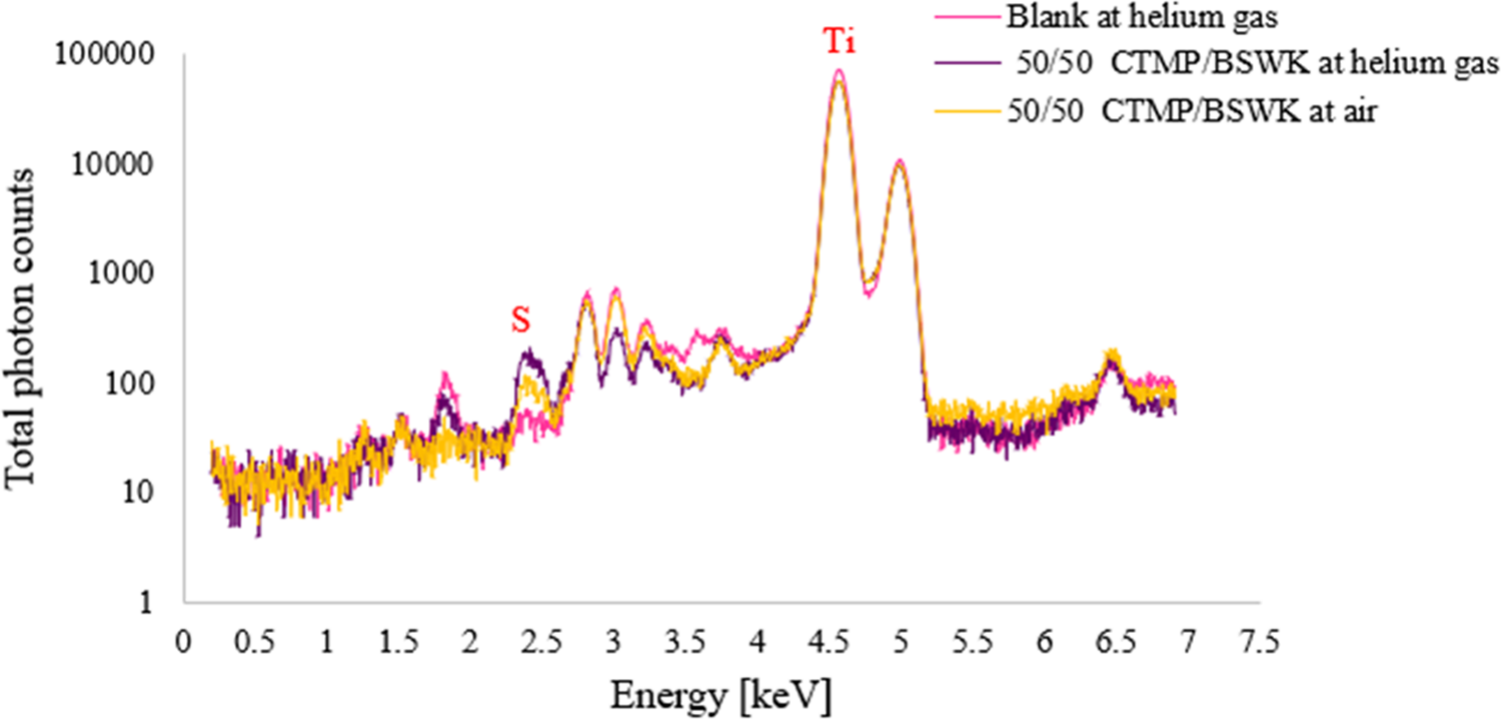
XRF analysis of CTMP and BSWK (50:50) using in helium gas and air through a 50 μm pinhole.

The sodium concentration for CTMP pulp samples was very low, making it difficult to detect the sodium peak with 10 and 50 μm pinholes. Therefore, we considered only measuring the sulfur from a 50 μm pinhole. As can be seen in Figure 6, handsheets of CTMP and BSWK (50:50) were investigated at 8 keV for 7 h in both helium gas and air environments. Our XRF setup clearly detected the sulfur peak (Kα_1_ 2.31 keV), especially when using the helium gas environment. Background noise around 2.3 keV was reduced by shielding the lead (Pb) Mα_1_-line signal with an aluminum pipe. Due to the small, irradiated area, low beam intensity, and low sodium concentration, the sodium peak was unclear in the spectrum. In addition, a polycapillary X-ray optic (not studied here) might be used as an alternative, which would provide a 150-fold increase in X-ray flux gain over a laboratory-prepared pinhole.^43^ Moreover, these considerations encouraged future measurements of sodium at a higher beam intensity in a vacuum environment at any synchrotron lab.

### XRF Setup in the Air Environment

**Figure 7:**
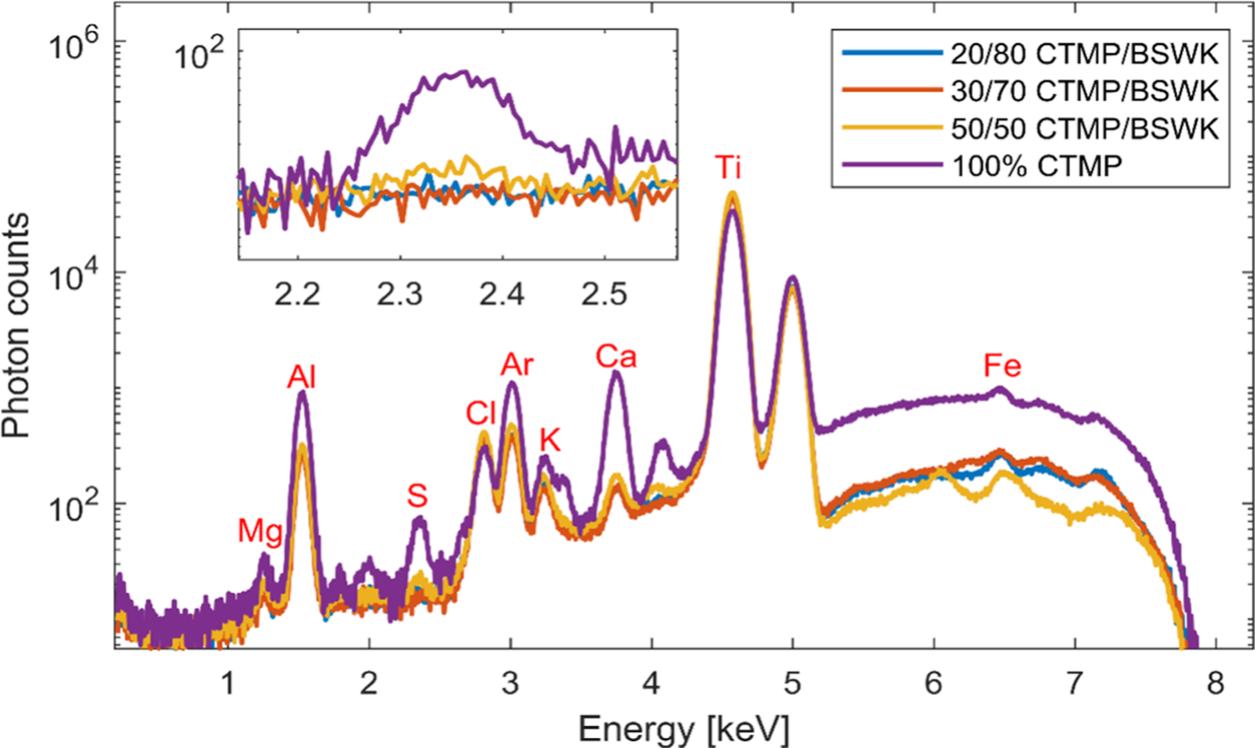
Analysis of single spots of 100% CTMP, 50:50, 30:70, and 20:80 CTMP/BSWK mixtures in an air environment using a 50 μm pinhole.

In spite of some background noise at this XRF setup, a single line scan of sulfur photon counts on CTMP handsheets with different percentages was performed in an air environment. The consideration of using in an air environment at laboratory setup was due to the consumption of helium gas that continues during use in the experiment that the balloon cylinder empties. Furthermore, the air absorption of sulfur is relatively smaller than that of sodium. An aluminum foil, however, was placed in front of the 50 μm pinhole to reduce the continuous X-rays from the tube. We scanned for 24 h at one spot point in single line scanning for 20 gm/m^2^ handsheets produced from 100% CTMP, 50:50 CTMP/BSWK, 30:70 CTMP/BSWK, and 20:80 CTMP/BSWK at 7 keV in an air environment. Figure 7 shows that the highest sulfur peak (Kα_1_ 2.31 keV) was observed for a 100% CTMP handsheet in comparison to 50–20% CTMP and BSWK mixtures. It should be noted that the background of the CTMP sample was corrected by a factor of three due to the difference in thickness between these samples. As the sulfonate group is covalently bound to the lignin at CTMP, it can be easily detected by XRF.

At Figure 7, spectra resolved Mg (Kα_1_ 1.25 keV) from the X-ray tube, Al (Kα_1_ 1.48 keV) from the Al filter, S (Kα_1_ 2.31 keV) and Cl (Kα_1_ 2.62 keV) are accepted from the sample, Ar (Kα_1_ 2.96 keV) from the atmosphere, Ca (Kα_1_ 3.69 keV) from the paper sample, and Ti (Kα_1_ 4.51 keV) and Fe (Kα_1_ 6.40 keV) from the set housing materials. Fluorescence X-ray intensity is related to the thickness of a thin sample sheet. As a result, the thickest sample, 100% CTMP, exhibited a strong Ca peak. If different elements are analyzed in the same surroundings, then the saturation depth will decrease as the element’s atomic number decreases. We do not correct for the difference in the thickness of the 100% CTMP sample compared to the other samples.

**Figure 8:**
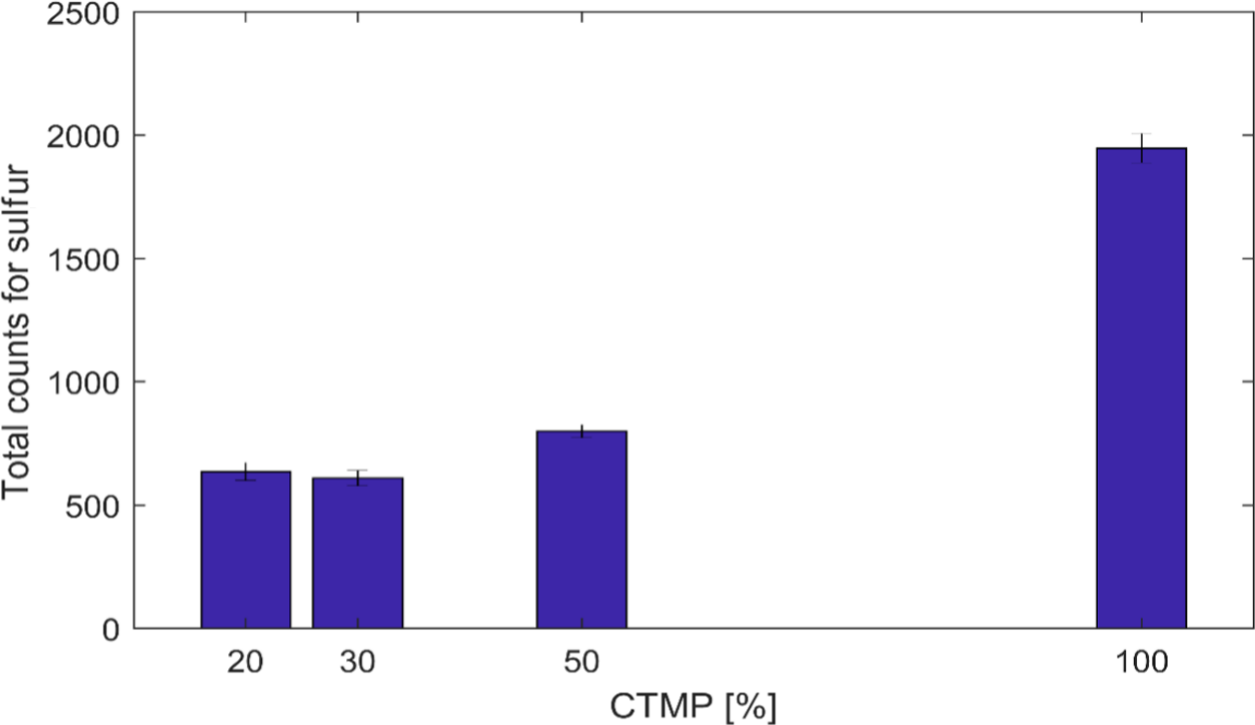
Integration of total photon counts for sulfur 100% CTMP, 50:50, 30:70, and 20:80 CTMP/BSWK mixtures in the air environment using a 50 μm pinhole.

In XRF analysis, the area of a peak for a given element is directly proportional to its concentration within the sample volume. In Figure 8, the highest total counts of sulfur photons were observed at a 100% CTMP handsheet when the sulfur peak from all spectra was integrated and plotted. As the CTMP pulp consistency decreased at different handsheets, the number of sulfur photons decreased. As a result, the sulfur concentration variance was too small in 20 and 30% CTMP paper sheets.

**Figure 9:**
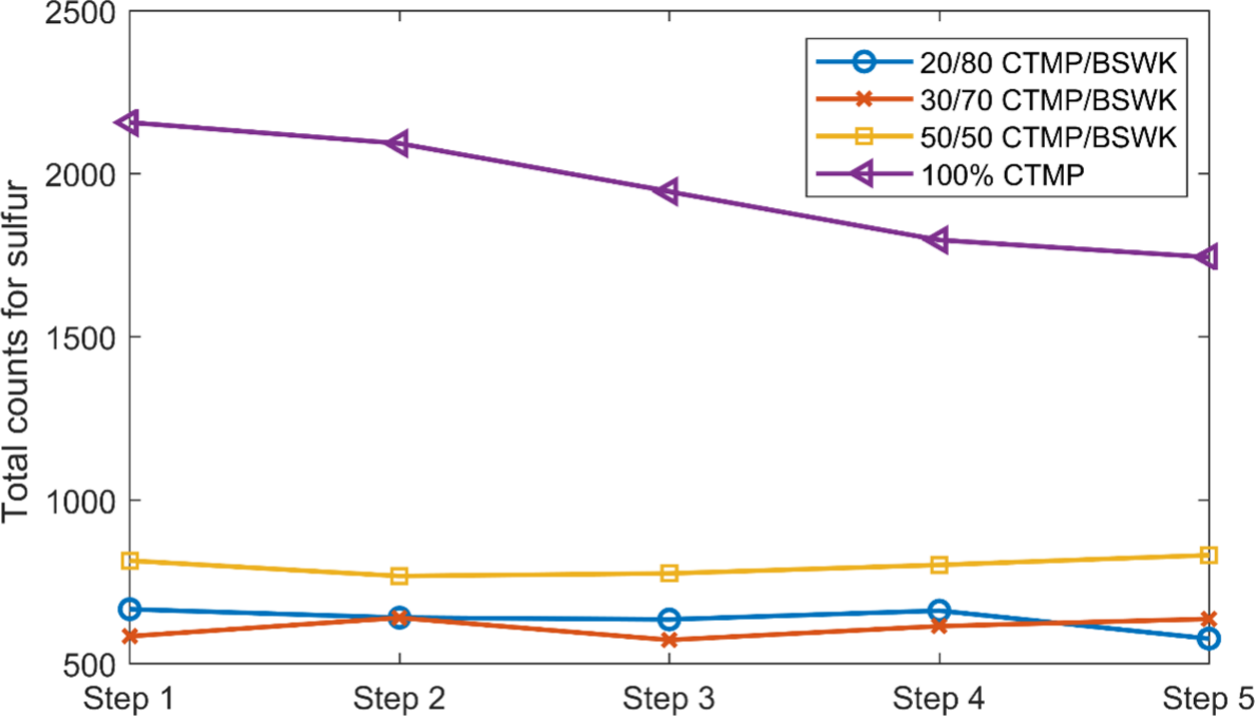
Photon counts for sulfur at five steps (75 μm steps). XRF analysis of 100% CTMP, 50:50, 30:70, and 20:80 CTMP/BSWK mixtures in an air environment using a 50 μm pinhole.

Additionally, a small variance was observed in the 50, 30, and 20% CTMP samples from the scanning of four different handsheets for five steps (300 μm distance), as shown in Figure 9.

Because of the unequal distribution of sulfonate in the wood chips during the impregnation state, each step in single line photon counts will not be the same. Paper samples with CTMP consistency below 50% already had a less sulfonated fiber. It was difficult to determine the homogeneity of sulfur photon counts due to the uneven distribution of sulfur in each paper sample based on single-point scanning. It might be the case that the point we choose was a very low sulfonated fiber. The difference between 30:70 and 20:80 at Figures 8 and 9 could therefore not be distinguished from significant differences. Moreover, the single-spot analysis for 24 h took into account random errors such as generators and X-ray tube stability, as well as counting statistics. It was suggested for the next industrial lab setup that we continue single-fiber analysis with a higher beam intensity, aiming for 10 μm resolution. In this case, it is more effective to use polycapillary X-ray optics to encapsulate between 10 and 20 μm for single-fiber analysis rather than XRF pinholes. Polycapillary gain will provide 150 times more X-ray flux than our handmade 50 μm pinhole, resulting in a substantial reduction in measurement time.^44^

## Conclusions

XRF imaging using energy-dispersive X-ray spectroscopy (ED-XRF) has the ability to image light elements, especially sulfur, in both helium gas and air environments. Our lab setup for XRF imaging is validated by spectral measurement of Seltin salt for sodium and CTMP handsheets for sulfur individually. Our results show, however, that it is possible to develop a direct X-ray fluorescence method to measure sulfur in an air atmosphere. It also suggests the possibly to measure sodium, in particular at higher concentration levels, if an helium gas environment is used. Our setup has the capability to measure sulfur homogeneity with a spatial resolution in the order of 100 μm but must be further improved to measure homogeneity on individual fiber level. We suggest X-ray capillary optics as a way to improve the spatial resolution, as well as a way to shorten the necessary measurement time. In future studies, we suggest validation of an improved setup against sulfur imaging measurements from synchrotron facilities before going to large industrial lab trials.

## Abbreviations

CMP: chemimechanical pulp
CTMP: chemithermomechanical pulp
HTCTMP: high-temperature chemithermomechanical pulp
ED-XRF: energy-dispersive X-ray fluorescence
NSSC: neutral sulfite semichemical
SEM: scanning electron microscope
BSWK: bleached softwood kraft
MCNP: Monte Carlo-*N*-particle radiation transport code
XOP: X-ray-oriented programs
FWHM: full width at half-maximum
LOD: limit of detection

## References

[ref1] Ferritsius O.; Moldenius S. In The Effect of Impregnation Method on CTMP Properties, International Mechanical Pulping Conference Proceedings; SPCI: Stockholm, Sweden, 1985; p 91.

[ref2] Kleppe P. J. Kraft pulping. Tappi 1970, 53, 35–47.

[ref3] Malkov S.; Tikka P.; Gullichsen J. Towards complete impregnation of wood chips with aqueous solutions. Paperi ja Puu–Paper Timber 2001, 83, 468–473.

[ref4] Robinson J. K. Measurement of kappa number variability on the fiber level. Tappi J. 2002, 1, 3–8.

[ref5] Malkov S.; Tikka P.; Gustafsson R.; Nuopponen M.; Vourinen T. Towards complete impregnation of wood chips with aqueous solutions. Part 5: Improving uniformity of kraft displacement batch pulping. Pap. Puu 2003, 85, 215–220.

[ref6] Wang Y.; Azhar S.; Lindström M. E.; Henriksson G. Stabilization of polysaccharides during alkaline pre-treatment of wood combined with enzyme-supported extractions in a biorefinery. J. Wood Chem. Technol. 2015, 35, 91–101. 10.1080/02773813.2013.875041.

[ref7] Tavast D.; Brännvall E. Increased pulp yield by prolonged impregnation in softwood kraft pulping. Nord. Pulp Pap. Res. J. 2017, 32, 14–20. 10.3183/npprj-2017-32-01-p014-020.

[ref8] Brännvall E.; Reimann A. The balance between alkali diffusion and alkali consuming reactions during impregnation of softwood. Impregnation for kraft pulping revisited. Holzforschung 2018, 72, 169–178. 10.1515/hf-2017-0078.

[ref9] Hellström L. M.; Gradin P. A.; Engstrand P.; Gregersen Ø. Properties of wood chips for thermomechanical pulp (TMP) production as a function of spout angle. Holzforschung 2011, 65, 805–809. 10.1515/HF.2011.087.

[ref10] Engstrand P.; Gradin P.; Hellström L.; Carlberg T.; Sandström P.; Liden J.; Egnell M. In Improved Refining Energy Efficiency in Thermo-Mechanical Pulping by Means of Collimated Wood Chipping from Solid Mechanics to Full Scale Evaluation, Paper Week Canada Conference, 2016.

[ref11] Höglund H. E.; Pettersson P. V. Chip Treatment (The Chips can be Steamed Prior to the Impregnation Process to Increase the Liquid Absorption). U.S. Patent US4,356,213A, 1982.

[ref12] Bengtsson G.; Simonson R.; Heitner C.; Beatson R.; Ferguson C. Chemimechanical pulping of birch wood chips, Part 2: Studies on impregnation of wood blocks using scanning electron microscopy and energy dispersive x-ray analysis. Nord. Pulp Pap. Res. J. 1988, 3, 132–138. 10.3183/npprj-1988-03-03-p132-138.

[ref13] Svensson E.; Engstrand P.; Htun M.; Svensson B. A better balance between shives content and light-scattering properties of TMP/CTMP by SO_2_ gas-phase impregnation prior to defibration. Nord. Pulp Pap. Res. J. 1994, 9, 167–171. 10.3183/npprj-1994-09-03-p167-171.

[ref14] Gorski D.; Hill J.; Engstrand P.; Johansson L. Reduction of energy consumption in TMP refining through mechanical pre-treatment of wood chips. Nord. Pulp Pap. Res. J. 2010, 25, 156–161. 10.3183/npprj-2010-25-02-p156-161.

[ref15] Atack D.; Heitner C.; Stationwala M. I. Ultra high yield pulping of eastern black spruce refiner mechanical and thermomechanical pulps (TMP)-pretreatment, properties energy consumption. Sven. Papperstidn. 1978, 81, 164–176.

[ref16] Gellerstedt G. The reaction of lignin during sulphite pulping. Sven. Papperstidn. 1976, 79, 537–543.

[ref17] Becker H.; Höglund H.; Tistad G. Frequency and temperature in chip refining. Pap. Puu 1977, 59, 123–130.

[ref18] Johansson L.; Peng F.; Simonson R. Effects of temperature and sulphonation on shear deformation of spruce wood. Wood Sci. Technol. 1997, 31, 105–117. 10.1021/es9601696.

[ref19] Engstrand P.; Hammar LÅ.; Htun M. In The Kinetics of Sulphonation Reactions on Norwegian Spruce, 3rd International Symposium of Wood and Pulping Chemistry, Vancouver, Canada. Canadian Pulp and Paper Association, 1985; pp 275–79.

[ref20] Lai Y.-Z.; Iwamida T. Effects of chemical treatments on ultra-high- yield pulping 1. Fiber separation. Wood Sci. Technol. 1993, 27, 195–203. 10.1007/BF00192816.

[ref21] Nelsson E. Improved Energy Efficiency in Mill Scale Production of Mechanical Pulp by Increased Wood Softening and Refining Intensity. PhD Thesis, Faculty of Science, Technology and media, Mid Sweden University, Sweden, 2016.

[ref22] Sandberg C.; Nelsson E.; Engberg B. A.; Berg J.; Engstrand P. Effects of chip pretreatment and feeding segments on specific energy and pulp quality in TMP production. Nord. Pulp Pap. Res. J. 2018, 33, 448–459. 10.1515/npprj-2018-3052.

[ref23] Persson E.; Norgren S.; Engstrand P.; Johansson M.; Edlund H. In Spruce HT-CTMP Revisited – A High Yield, Energy Efficient Pulp for Future Products, 11th Fundamental Mechanical Pulp Research Seminar; Norrköping: Sweden, 2019.

[ref24] Heazel T. E. Cell Wall Sulphur Distribution in Sulphonated Southern Pine Latewood. PhD Thesis, Institute of Paper Chemistry, Lawrence University, Appleton, Wisconsin, USA, 1988.

[ref25] Vikstrom B.; Hammar L. A. In Softening of Spruce Wood during Sulphite Pulping and its Relevance for the Character of High-Yield Pulps, Proceedings of the International Symposium on Wood and Pulping Chemistry, Stockholm, 1981; Vol. 5, p 112.

[ref26] Axelson P.; Simonson R. Thermomechanical pulping with low addition of sulphite: influence of the preheating temperature at mild sulphite treatment of spruce chips prior to defibration. Pap. Puu 1982, 64, 729–733.

[ref27] Nelsson E.; Sandberg C.; Hildén L.; Daniel G. Pressurised compressive chip pre-treatment of Norway spruce with a mill scale Impressafiner. Nord. Pulp Pap. Res. J. 2012, 27, 56–62. 10.3183/npprj-2012-27-01-p056-062.

[ref28] Almkvist G.; Persson I. Distribution of iron and sulfur and their speciation in relation to degradation processes in wood from the Swedish warship Vasa. New J. Chem. 2011, 35, 1491–1502. 10.1039/c1nj20056a.

[ref29] Sixta H. Handbook of Pulp; WILEY-VCH Verlag GmbH & Co, KGaA: Weinheim, 2006; Vol. 1, p 154, ISBN 3-527-30999-3.

[ref30] Abou-Ras D.; Caballero R.; Fischer C. H.; Kaufmann C. A.; Lauermann I.; Mainz R.; Mönig H.; Schöpke A.; Stephan C.; Streeck C.; Schorr S.; et al. Comprehensive comparison of various techniques for the analysis of elemental distributions in thin films. Microsc. Microanal. 2011, 17, 728–751. 10.1017/S1431927611000523. 21906418

[ref31] Fröjdh C.; Norlin B.; Frojdh E. Spectral X-ray imaging with single photon processing detectors. Journal of Instrum. 2013, 8, C02010 10.1088/1748-0221/8/02/C02010.

[ref32] Reza S.; Norlin B.; Thim J.; Fröjdh C. Non-destructive method to resolve the core and the coating on paperboard by spectroscopic x-ray imaging. Nord. Pulp Pap. Res. J. 2013, 28, 439–442. 10.3183/npprj-2013-28-03-p439-442.

[ref33] Norlin B.; Reza S.; Fröjdh C.; Nordin T. Precision scan imaging for paperboard quality inspection utilizing X-ray fluorescence. J. Instrum. 2018, 13, C01021 10.1088/1748-0221/13/01/C01021.

[ref34] Saka S.; Thomas R. J.; Gratzl J. S.; Abson D. Topochemistry of delignification in Douglas-fir wood with soda, soda-anthraquinone and karft pulping as determined by SEM-EDXA. Wood Sci. Technol. 1982, 16, 139–153. 10.1007/BF00351099.

[ref35] Handheld XRF: How it works. BRUKER webpage, 2021. https://www.bruker.com/products/x-ray-diffraction-and-elemental-analysis/handheld-xrf/how-xrf-works.html.

[ref36] Rahman H.; Lindström M. E.; Sandstöm P.; Salmén L.; Engstrand P. The effect of increased pulp yield using additves in the softwood kraft cook on the physical properties of low-grammage handsheets. Nord. Pulp Pap. Res. J. 2017, 32, 317–323. 10.3183/npprj-2017-32-03-p317-323.

[ref37] Andersson P. A Dynamic Na/S Balance of Kraft Pulp Mill: Modelling and Simulation of a Kraft Pulp Mill Using WinGEMS, Exam Work. Department of Engineering and Chemical science, Chemical Engineering. Master Thesis, Karlstad University, Sweden, 2014.

[ref38] Fevang P. Seltin-Far from the healthy salt. Tidsskrift Den norske legeforening 2009, 129, 1123 10.4045/tidsskr.09.0537. 19488100

[ref39] Werner C. J. MCNP User’s Manual, Code Version 6.2. Los Alamos National Laboratory Report, LA-UR-17-29981, 2017.

[ref40] Lider V. X-ray fluorescence imaging, Instruments and Methods of Investigation. Uspekhi Fizicheskikh Nauk, Russian Academy of Sciences. Phys.-Usp. 2018, 61, 980 10.3367/UFNe.2017.07.038174.

[ref41] Siwen A. X-ray Fluorescence Spectrometry for Environment Applications. Licentiate Thesis, Faculty of Science, Technology and media, Mid Sweden University, Sweden, 2020; ISSN 1652-8948.

[ref42] Medeiros I. M. M. A.; Zamboni C. B.; Da Cruz M. T. F.; Morel J. C. O.; Park S. W. In Semi-Parametrical NAA Method for Paper Analysis, International Nuclear Atlantic Conference, INAC Santos, SP, Brazil, 2007.

[ref43] Polycapillary X-ray Optics for Micors XRF and XRD. XOS webpage 2020. PolycapillaryOptics_Datasheet_ext.5.9.2018.pdf (xos.com).

[ref44] Siwen A. Spectroscopic and Microscopic X-ray Fluorescence Analysis for Environmental and Industrial Applications. PhD Thesis, Faculty of Science, Technology and media, Mid Sweden University, Sweden, 2022; ISBN 978-91-89341-66-1.

